# Involvement of AMPK in regulating the degradation of MAD2B under high glucose in neuronal cells

**DOI:** 10.1111/jcmm.13046

**Published:** 2016-12-13

**Authors:** Xianfang Meng, Guangpin Chu, Chen Ye, Hui Tang, Ping Qiu, Yue Hu, Man Li, Chun Zhang

**Affiliations:** ^1^ Department of NeurobiologySchool of Basic Medical SciencesTongji Medical CollegeHuazhong University of Science and TechnologyWuhanChina; ^2^ Institute of Brain ResearchHuazhong University of Science and TechnologyWuhanChina; ^3^ Department of NephrologyUnion HospitalTongji Medical CollegeHuazhong University of Science and TechnologyWuhanChina

**Keywords:** MAD2B, high glucose, AMPK, protein degradation, neuron

## Abstract

Although our recent study has demonstrated that mitotic spindle assembly checkpoint protein (MAD2B) mediates high glucose‐induced neuronal apoptosis, the mechanisms for MAD2B degradation under hyperglycaemia have not yet been elucidated. In this study, we first found that the activation of adenosine 5′‐monophosphate (AMP)‐activated protein kinase (AMPK) was decreased in neurons, accompanied with the increased expression of MAD2B. Mechanistically, we demonstrated that activation of AMPK with its activators such as AICAR and metformin decreased the expression of MAD2B, indicating a role of AMPK in regulating the expression of MAD2B. Moreover, activation of AMPK prevented neuronal cells from high glucose‐induced injury as demonstrated by the reduced expression of cyclin B1 and percentage of apoptosis as detected by TUNEL. We further found that when total protein synthesis was suppressed by chlorhexidine, the degradation of MAD2B was slower in high glucose‐treated neurons and was mainly dependent on the ubiquitin–proteasome system. Finally, it was indicated that high glucose inhibited the ubiquitination of MAD2B, which could be reversed by activation of AMPK. Collectively, this study demonstrates that AMPK acts as a key regulator of MAD2B expression, suggesting that activation of AMPK signalling might be crucial for the treatment of high glucose‐induced neuronal injury.

## Introduction

Diabetes is a systemic disease mainly characterized by chronic hyperglycaemia 1. Its resultant increase in morbidity and mortality is mainly due to its complications such as foot ulcer/gangrene, poor wound healing, stroke, renal diseases and eye diseases 2, 3. Therefore, its resultant complications are becoming main public health problems. Accumulating data in the last decade have suggested that diabetes mellitus also malignantly affects central nervous system (CNS). The abnormalities such as temporal lobe sclerosis, declines in white matter volume as well as decreased grey matter volumes, are common in diabetic brains 4, 5. All these structural changes in brains will lead to cognitive impairment, specifically affected learning and memory 6. Moreover, recent data suggest that diabetes is a predisposing factor for Alzheimer's disease. However, its mechanisms remain unclear and therapies are limited.

Identifying the pathophysiological mechanisms underlying cognitive impairment in diabetes is of great interest and importance. It has been indicated that degeneration and apoptosis of hippocampal and frontal cortical neurons induced in diabetes contribute to the deficit of learning and memory of diabetic rodents 7. It has been suggested that anti‐apoptosis using fish oil, berberine and vitamin D prevents neuronal apoptosis and improves learning and memory 8, 9. However, the mechanism of neuron apoptosis in diabetes is not fully elucidated.

Recently, it has been reported that cyclin B plays a vital role in the survival of post‐mitotic neurons 10. Cyclin B has been found to accumulate in degenerating brain areas in Alzheimer's disease and stroke 11. The increased expression of cyclin B leads to terminally differentiated neurons aberrantly re‐entering the cell cycle, which will result in neuronal death *via* apoptosis 12. It has been shown that the anaphase‐promoting complex/cyclosome (APC/C), an E3 ubiquitin ligase that is highly active in neurons, has a basic role in regulating the degradation of cyclin B 13. Anaphase‐promoting complex/cyclosome activity is regulated by its activators (Cdc20 and Cdh1) and its inhibitor (mitotic spindle assembly checkpoint protein, MAD2B) 14, 15. It has been shown that Cdc20 and Cdh1 play vital roles in post‐mitotic neurons, which regulate neuronal survival, differentiation, axonal growth and synaptic development through APC/C 16, 17, 18. However, less research work focuses on the role of MAD2B in neurons.

In our previous study, we found that MAD2B is mainly expressed in neurons of CNS 19. Moreover, we found that high glucose induced the expression of MAD2B and accumulation of cyclin B1 *in vivo* and *in vitro*. Inhibition of MAD2B expression prevented neurons from apoptosis induced by high glucose 20. These results indicated that MAD2B plays an important role in regulating neuronal apoptosis in diabetes. However, it is still unknown the mechanisms which regulate the degradation of MAD2B under high glucose. Therefore, in the present study we explored the possible pathway involved in modulating the degradation of MAD2B.

## Materials and methods

### Primary neuronal cell culture

Primary neuronal cell culture and treatment with glucose were performed as described previously 19. Briefly, primary cultures of rat cortical neurons were prepared from the of E17–E18 Sprague Dawley rat embryos. The cells were dissociated in Neurobasal medium (Gibco Invitrogen, Grandland, NY, USA), supplemented with B27 (1:50 dilution; Gibco Invitrogen), 0.5 mM glutamine, 25 μM glutamate, 50 μg/ml gentamycin and 25 mM basal glucose. The cells were plated in six‐well plates coated with poly‐D‐lysine (0.1 mg/ml). The cultures were maintained in a humidified incubator with 5% CO_2_/95% air at 37°C for at least 6 days. This medium was subsequently given half‐changes every 3 days.

### Cellular treatment

The following pharmacological treatments were used: high glucose (50 mM), MG132 (20 μM), NH_4_CL (25 mM), 3‐MA (5 mM), AICAR (0.5 mM), metformin (10 mM), actinomycin D (A‐D, 1.5 μM) or cycloheximide (CHX, 10 μg/ml) (all the reagents were from Tocris Biosciences, Minneapolis, MN, USA). At the end of the treatments, cells were washed in phosphate‐buffered saline (PBS) and lysed in ice‐cold lysis buffer supplemented with protease inhibitor cocktail (Sigma‐Aldrich, Saint Louis, MO, USA) for following detection. Western blot analyses were performed as previously described 21. Primary antibodies to MAD2B (1:1000 dilution; Rockland Immunochemicals Inc., Limerick, PA, USA), total AMPKα and phospho‐AMPKα (Thr172) (1:1000 dilution; Cell Signaling Technology, Inc., Boston, MA, USA), cyclin B1 (1:300 dilution; Proteintech, Inc., Wuhan, Hubei, PRC) and secondary antibodies horseradish peroxidase‐labelled antimouse IgG or anti‐rabbit IgG (1:6000 dilution; Santa Cruz Biotechnology, Santa Cruz, CA, USA) were used in this study. To document the loading controls, the membrane was reprobed with a primary antibody against housekeeping protein β‐actin.

### Immunoprecipitation

Co‐immunoprecipitation experiments were performed with Protein A/G PLUS‐Agarose (Santa Cruz Biotechnology) following the manufacturer's instructions. Briefly, to minimize non‐specific binding, 200 μg of protein lysates from treated neurons was precleared using a control agarose resin. The precleared lysates were incubated with 1 μg primary anti‐ubiquitin antibody for 1 hr at 4°C, and then 20 μl of resuspended volume of Protein A/G PLUS‐Agarose was added and incubated overnight. The resin was separated by centrifugation, washed three times with ice‐cold lysis buffer and then boiled in SDS sample buffer. Immunoblot analysis was performed with the primary antibody anti‐MAD2B, horseradish peroxidase‐conjugated antibody to mouse (1:20,000 dilution; Promega, Madison, WI, USA) and an enhanced chemiluminescence system (ECL, Amersham).

### TUNEL staining

According to the manufacturer's instructions apoptosis was detected with the TMR red *In Situ Cell Death Detection Kit* (Roche, Basel, CH, Switzerland). Terminal transferase was omitted as a negative control. Cells were exposed to DNase I prior to the assay (10 min., Roche) to provide a positive control. TUNEL‐positive cells were counted by an experimenter who was blind to the treatment groups.

### Statistical analysis

All data are presented as means ± S.E.M. *t*‐test was used to compare experimental groups to control values. Comparisons between multiple groups were made using Student–Newman–Keuls (SNK) test. Statistical significance was determined as *P* < 0.05.

## Results

### Expression of MAD2B related with the phosphorylation of AMPK

**Figure 1:**
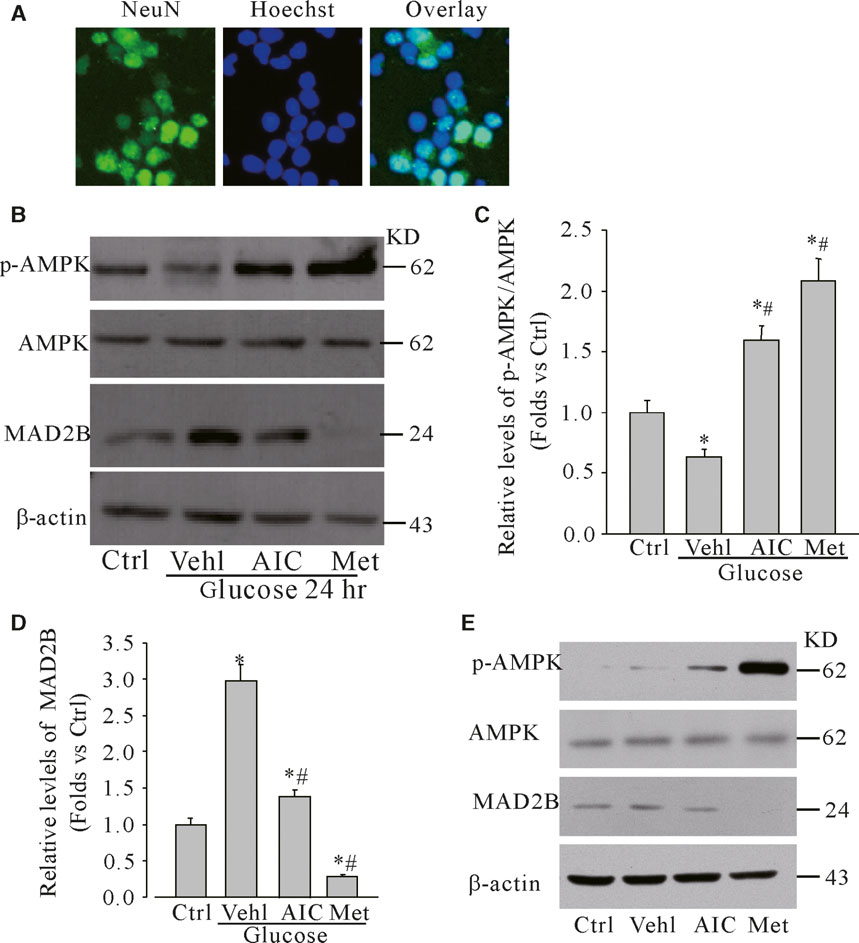
Expression of MAD2B related with the phosphorylation of AMPK. (**A**) Neurons cultured for at least 6 days were stained with NeuN and Hoechst showing the purity of primary neuronal cells. (**B**) Western blot analysis of the phosphorylation levels of AMPK and MAD2B protein expression from untreated, 50 mM glucose‐treated, AICAR‐treated and metformin‐treated cortical neurons. (**C** and **D**) Summarized data showing the band intensities for p‐AMPK and MAD2B, respectively. (**E**) Western blot analysis showing the effects of AICAR and metformin on the basal level of MAD2B in neuronal cells. Ctrl: control; Vehl: vehicle; AIC: AICAR; Met: metformin. *n* = 5, *P < 0.05 *versus* control; #*P* < 0.05 *versus* glucose.

**Figure 2:**
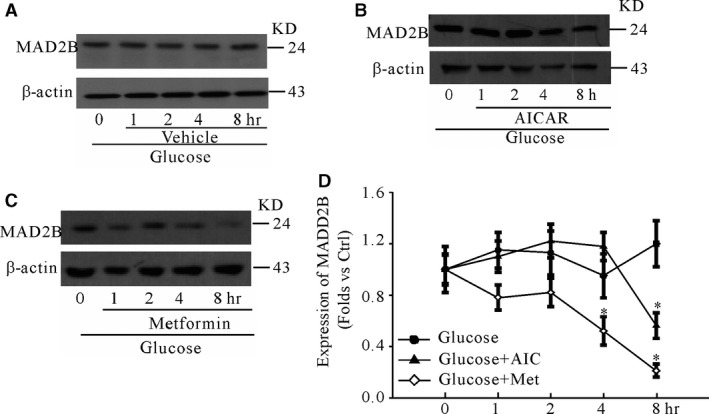
Activation of AMPK prevents MAD2B expression under high glucose. Cortical neurons were treated with 50 mM glucose for 24 hrs and then incubated with metformin (or AICAR) for different time‐points. (**A**–**C**) Western blot analysis showing the effects of metformin and AICAR on the expression of MAD2B under 50 mM glucose. (**D**) Summarized data. *n* = 5, *P < 0.05 *versus* 0 hr.

We first characterized the purity of primary neuronal cells. It was found that almost over 90% of cells were neurons as shown in Figure 1A, which indicated that the primary neuronal cells could be used for following research work. Then, we found that under high glucose, the induction of MAD2B expression was accompanied with the decreased phosphorylation level of AMPK. Pretreatment with AICAR or metformin, activators of AMPK, the expression levels of MAD2B induced by high glucose were significantly inhibited in cultured cortical neurons (Fig. 1B–D), showing that activation of AMPK significantly decreased the protein levels of MAD2B under high glucose. We further found that at the normal culture condition metformin significantly reduced the expression of MAD2B (Fig. 1E). To further explore whether AMPK was involved in affecting high glucose‐induced expression of MAD2B, cortical neurons were treated with high glucose for 24 hrs and then incubated with activators of AMPK (AICAR and metformin) for different time‐points. It was shown that activation of AMPK significantly decreased the protein levels of MAD2B under high glucose (Fig. 2A–D).

### Activation of AMPK reduced neuronal injury induced by high glucose

**Figure 3:**
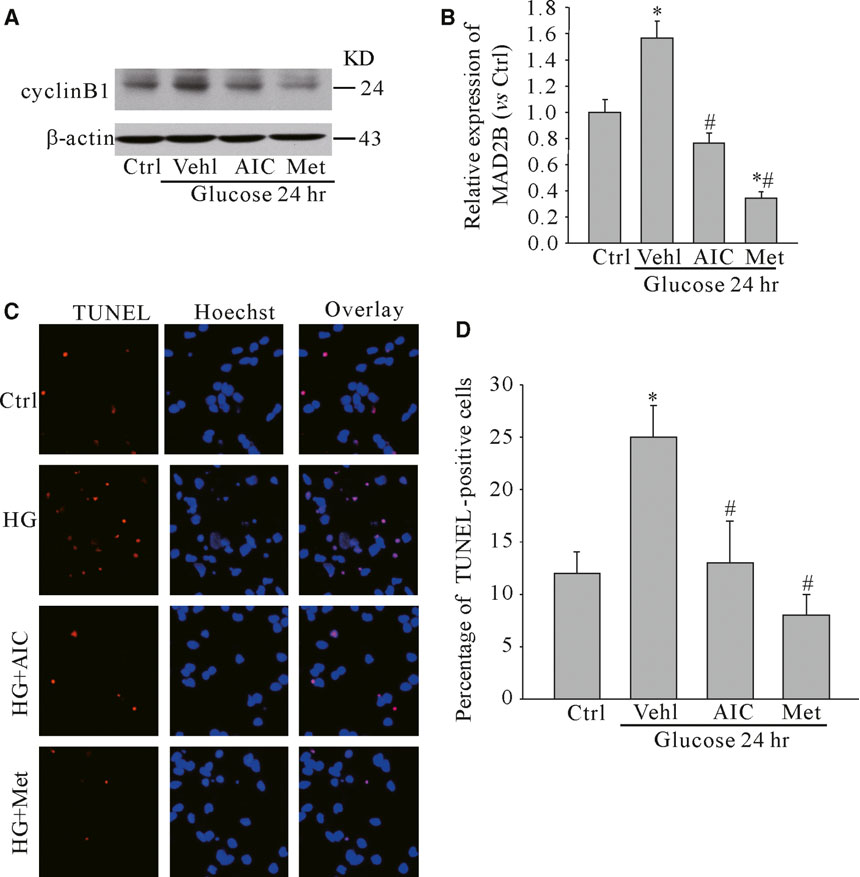
Activation of AMPK reduced neuronal injury induced by high glucose. (**A** and **B**) Represent gel images and summarized data showing the effects of AICAR and metformin on cyclin B1 expression under high glucose in cultured neurons. (**C** and **D**) Represent images and summarized data showing the effects of AICAR and metformin on neuronal apoptosis. Ctrl: control; Vehl: vehicle; HG: high glucose (50 mM); AIC: AICAR; Met: metformin. *n* = 5, *P < 0.05 *versus* Control; #*P* < 0.05 *versus* glucose.

To clarify the effects of AMPK on neuronal injury under high glucose, cyclin B1 was detected as its accumulation affects neuronal survival 10. It was indicated that activation of AMPK with its activator such as AICAR and metformin decreased the expression of cyclin B1 under high glucose (Fig. 3A and B). Moreover, it was found that AICAR and metformin also reduced neuronal apoptosis induced by high glucose as detected by TUNEL assay (Fig. 3C and D).

### Degradation of MAD2B was inhibited by high glucose in cortical neurons

**Figure 4:**
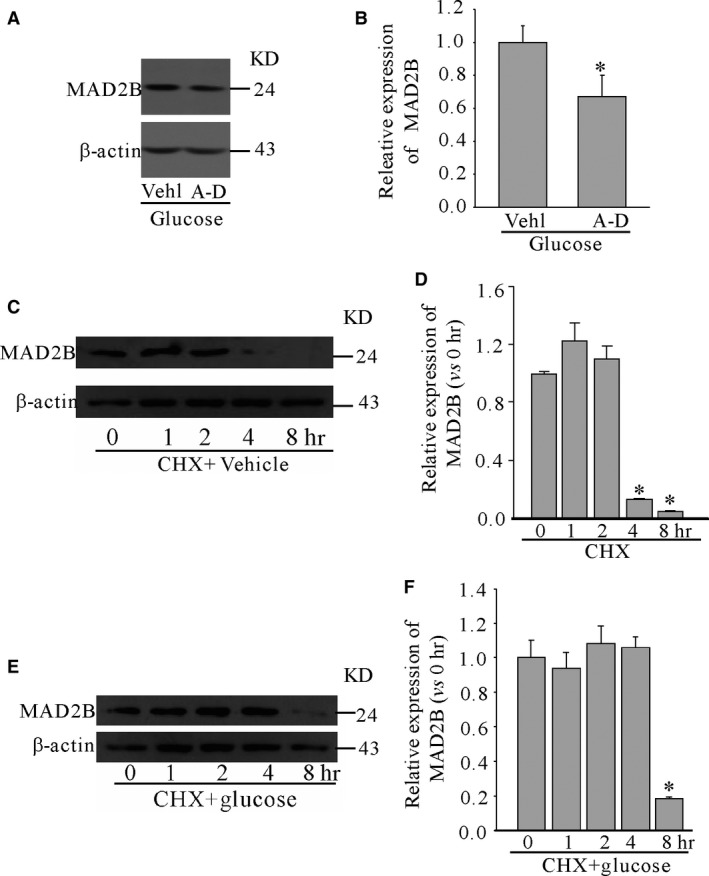
Degradation of MAD2B was inhibited by high glucose in cortical neurons. (**A** and **B**) Represent gels and summarized data showing the effects of transcription inhibitor actinomycin D on the expression of MAD2B. Neurons were pretreated with actinomycin D (1.5 μM) for 30 min., and then incubated with 50 mM glucose for 12 hrs. *P < 0.05 *versus* vehicle. (**C** and **E**) Western blot analysis of the effects of CHX on the expression of MAD2B under normal culture medium and 50 mM glucose, respectively. (**D** and **F**) Summarized data showing the band intensities for MAD2B under normal and high glucose‐treated neurons, respectively. *n* = 5, *P < 0.05 *versus* 0 hr.

To characterize how high glucose affects the expression of MAD2B, neuronal cells were pretreated for 30 min with actinomycin D (one of transcription inhibitors) and then incubated with 50 mM glucose for 12 hrs. We found that the expression of MAD2B was still at the high level although actinomycin D inhibited part of its expression (Fig. 4A and B), which suggested that in addition to the transcriptional level, post‐translational modification also plays a vital role in regulating the expression of MAD2B. Then, CHX, an inhibitor of protein biosynthesis in eukaryotic organisms, was incubated for different time‐points. It was shown that the levels of MAD2B began significantly decreased after incubation with CHX for 4 hrs at the normal culture condition (Fig. 4C and D). However, when in cortical neurons first treated with high glucose for 24 hrs, and then incubated CHX, the expression of MAD2B was still at higher levels at 4 hrs compared with the control (Fig. 4E and F). These results suggested that high glucose prevented the degradation of MAD2B protein.

### Degradation of MAD2B was dependent on ubiquitin–proteasome pathway

**Figure 5:**
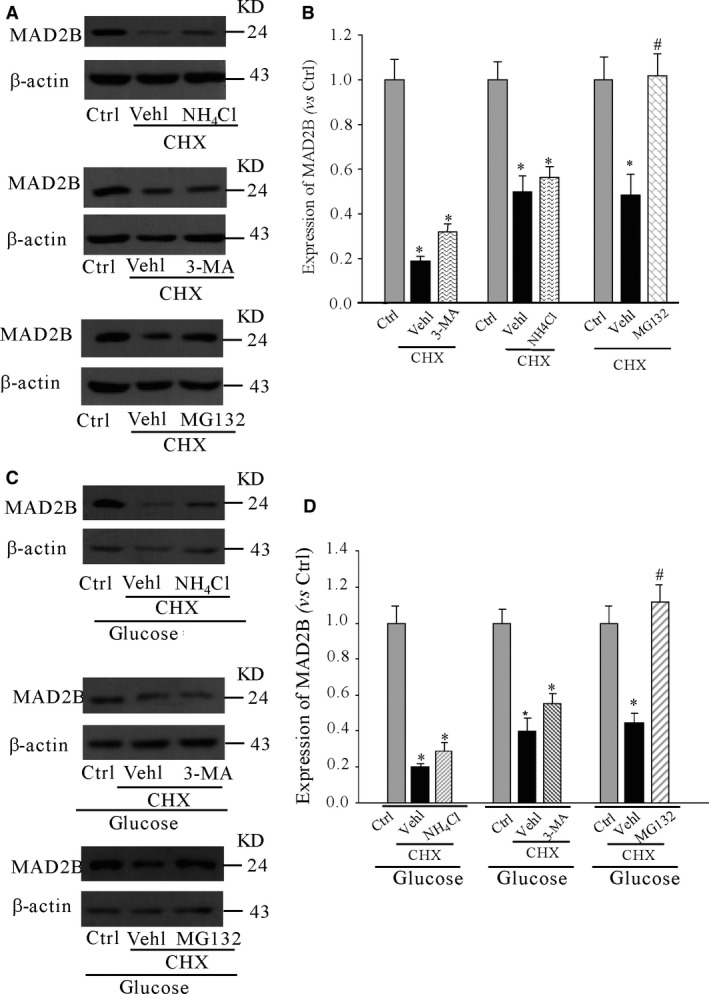
Degradation of MAD2B is dependent on ubiquitin–proteasome pathway. Cortical neurons were pretreated with 3‐MA (5 mmol/l), NH4Cl (5 mmol/l) or MG‐132 (20 μM) under normal or high glucose‐treated condition. (**A** and **C**) Western blot analysis of the effects of 3‐MA, NH4Cl and MG‐132 on the expression of MAD2B under normal culture medium and 50 mM glucose, respectively. (**B** and **D**) Summarized data showing the band intensities for MAD2B. Ctrl control; Vehl vehicle; CHX: cycloheximide. *n* = 5, *P < 0.05 *versus* control; #*P* < 0.05 *versus* glucose.

Next, to assess mechanisms of the degradation of MAD2B, we first detected the possible pathway under normal culture medium. It was shown that pretreatment of 3‐MA (5 mmol/l), a specific autophagic inhibitor, did not inhibit the degradation of MAD2B for 8 hrs. Moreover, NH_4_Cl (5 mmol/l) also showed the similar results. However, we found that MG132, as a peptide aldehyde effectively blocking the proteolytic activity of proteasome complex, prevented the degradation of MAD2B. All these results suggested that the degradation of MAD2B was mainly through ubiquitin–proteasome pathway, but not autophagy lysosomal pathway under normal culture in cortical neurons (Fig. 5A and B). Then, we elucidated the degradation mechanism of MAD2B under high glucose. Our result showed that MG132‐treated cells showed a more obvious increment of MAD2B protein level compared with MG132‐untreated cells, indicating that ubiquitination–proteasome system possibly also participates in the regulation of the stability of MAD2B protein under high glucose in neurons (Fig. 5C and D).

### AMPK promoted the degradation of MAD2B through ubiquitylation

**Figure 6:**
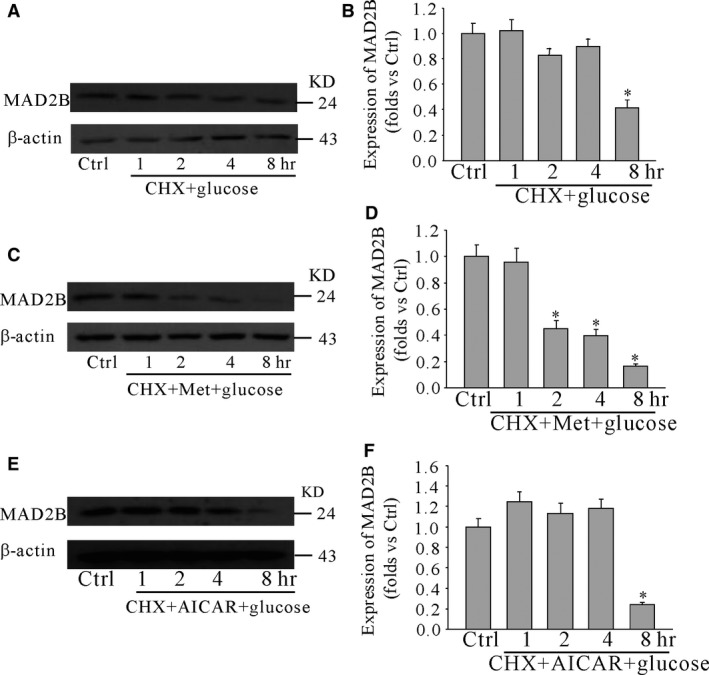
AMPK promoted the degradation of MAD2B. Cortical neurons were treated with metformin (or AICAR) for 30 min. and then treated with glucose for different time‐points. (**A**,** C**,** E**) Western blot analysis of the expression of MAD2B. (**B**,** D** and **F**) Summarized data showing the band intensities for MAD2B. Ctrl control; Vehl vehicle; CHX: cycloheximide; Met: metformin. *n* = 5, *P < 0.05 *versus* 0 hr.

**Figure 7:**
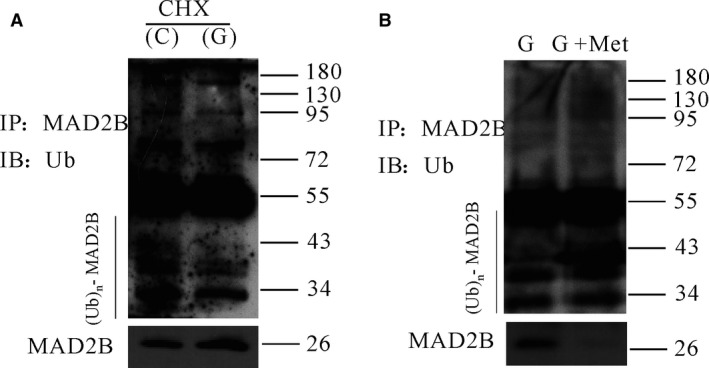
Metformin promoted the degradation of MAD2B through ubiquitylation. Cortical neurons were pretreated with CHX or Met for 30 min., then treated with high glucose for 4 hrs. (**A**) Western blot analysis showed the effects of high glucose on the ubiquitylation levels of MAD2B under high glucose. (**B**) Western blot analysis showed the effects of metformin on ubiquitylation levels of MAD2B. C: control; G: glucose; Met: metformin.

To further detect the mechanisms of AMPK regulating the expression of MAD2B, cortical neurons were pretreated with CHX and AICAR (or metformin) for 30 min. and then incubated with high glucose for different time‐points. It was shown that AICAR and metformin promoted the degradation of MAD2B (Fig. 6). To examine how AMPK was involved in the degradation of MAD2B, we first evaluated whether high glucose affected MAD2B ubiquitylation. The results of immunoprecipitation showed that high glucose inhibited MAD2B ubiquitylation. However, AMPK activator metformin increased MAD2B ubiquitylation (Fig. 7).

## Discussion

The major goal of this study was to address the degradation mechanisms of MAD2B under high glucose in neurons. Using cultured primary neurons, we first found that the expression of MAD2B was related with the phosphorylation level of AMPK. Moreover, activation of AMPK inhibited high glucose‐induced expression of MAD2B. We also showed that high glucose mainly increased the expression of MAD2B through preventing its degradation. Furthermore, activation of AMPK promoted MAD2B degradation through regulating its ubiquitylation. We thus propose one mechanism by which AMPK is involved in regulating the degradation of MAD2B under high glucose in neuronal cells, and activation of AMPK promotes the degradation of MAD2B.

MAD2B is on chromosome 1p36 and homologous to the spindle checkpoint gene MAD2 (MAD2L1), which plays an important role in aneuploidy, one important character of the majority of human cancers 14. MAD2B is a key component of a surveillance system that delays anaphase until all chromosomes are correctly oriented. Defects in this mitotic checkpoint are known to contribute to genetic instability, that is numerical and structural aberrations of chromosomes 22. It was reported that up‐regulated MAD2B expression in colon tumours had significantly higher numbers of aberrant mitotic figures (anaphase bridges), an indication of chromosomal instability. Elevated expression of MAD2B was significantly correlated with reduced patient survival 22. Moreover, it was also shown that MAD2B is overexpressed in human glioma, with depletion enhancing sensitivity to ionizing radiation 23. Furthermore, other research work has shown that suppression of MAD2B enhances cisplatin sensitivity in ovarian clear cell carcinoma cells 24. Thus, all these results demonstrate that MAD2B playing an important role in regulating cell cycle correlates with bad prognosis in cancers.

Further research indicated that MAD2B is an inhibitor of APC/C 25. APC/C is vital for neurons to maintain the state of differentiation. It is well known that neurons are permanently blocked their capacity to proliferate once they are differentiated. They usually are in a quiescent state in the adult nervous system 26. However, when neurons are under insults such as neurotrophic factor deprivation, activity withdrawal, DNA damage, oxidative stress, and excitotoxicity, they will re‐express cell cycle markers and try to re‐enter cell cycle 27, 28. Once neurons re‐enter cell cycle, most of them will ultimately die 29, 30. APC/C in neurons prevents the aberrant re‐entry of post‐mitotic neurons into the cell cycle 31 and plays a vital role for neuronal survival. However, whether MAD2B is expressed in neurons or plays an important role in keeping neuronal survival is still unknown. In our previous study, we found that MAD2B was widely expressed in cortical and hippocampal neurons 19. Moreover, we found that under high glucose, the expression of MAD2B was increased in neurons and played an important role for accumulating of cyclin B1 20. Therefore, it is important to investigate the mechanisms regulating the degradation of MAD2B under high glucose.

It has been reported that high glucose reduced AMPK phosphorylation and vice versa 32. Activated protein kinase is the key energy sensor and regulator for metabolic pathology 33. It is found that AMPK inactivation is involved in high glucose‐induced mitochondrial dysfunction and insulin resistance in primary cortical neurons and neuroblastoma cells, as well as in cerebral cortex of db/db mice 34. Therefore, we hypothesize that AMPK may be involved in regulating the expression of MAD2B under high glucose. We first successfully demonstrated that in primary neuronal cells high glucose reduced AMPK phosphorylation accompanied by increased expression of MAD2B. Then, we pretreated neurons with AMPK activators such as AICAR and metformin before adding glucose in the culture medium. The results showed that AMPK activators prevented the expression of MAD2B under high glucose. We may speculate that the increased expression of MAD2B is in conjunction with the activation of AMPK.

Then, we examined how AMPK affects the expression of MAD2B. We first detected that the degradation mechanism of MAD2B under normal and high‐glucose culture condition. We treated primary cortical neurons with NH4Cl, 3‐MA or MG132, and determined the protein levels of MAD2B. We showed that MG132 significantly increased MAD2B protein levels as compared to vehicle‐treated neurons. These findings suggest that proteasomal degradation plays an important role in the regulation of MAD2B in neurons. Moreover, it was found that activation of AMPK promoted the degradation of MAD2B under high glucose. Thus, it can be assumed that AMPK is involved in regulating the expression of MAD2B at the protein level in neurons. Our further findings showed that high glucose prevented the ubiquitination of MAD2B. Ubiquitination is a post‐translational modification that targets proteins for degradation 35. In the present study, we also found that AMPK activator metformin increased the ubiquitination of MAD2B.

Taken together, the present study shows that high glucose decreases the degradation of MAD2B, whose degradation is mainly dependent on ubiquitin–proteasome system. Moreover, activation of AMPK promotes the ubiquitination and degradation of MAD2B. Our results provide new insights underlying hyperglycaemia and cognition defect. Therefore, pharmacological targeting of AMPK may be a promising strategy in prevention against hyperglycaemia‐induced neuronal apoptosis and cognitive impairment in CNS.

## Disclosure statement

All authors have nothing to disclose any further actual or potential conflict of interest including financial, personal or other relationships with other people or organizations related to the research covered in this article.
